# “The midwife helped me ... otherwise I could have died”: women’s experience of professional midwifery services in rural Afghanistan - a qualitative study in the provinces Kunar and Laghman

**DOI:** 10.1186/s12884-020-2818-1

**Published:** 2020-03-06

**Authors:** Trude Thommesen, Hallgeir Kismul, Ian Kaplan, Khadija Safi, Graziella Van den Bergh

**Affiliations:** 1 Centre for International Health, Department for Global Public Health and Primary Health Care, University of Bergen, Bergen, Norway; 2 Norwegian Afghanistan Committee, Kabul, Afghanistan; 3 Western Norway University of Applied Sciences, Bergen, Norway

**Keywords:** Midwives, Afghanistan, Maternal health, women’s experience of midwifery care

## Abstract

**Background:**

Afghanistan has one of the world’s highest maternal mortality ratios, with more than 60% of women having no access to a skilled birth attendant in some areas. The main challenges for childbearing Afghan women are access to skilled birth attendance, emergency obstetric care and reliable contraception. The NGO-based project *Advancing Maternal and Newborn Health in Afghanistan* has supported education of midwives since 2002, in accordance with the national plan for midwifery education.

The aim of this study is to explore women’s experiences of professional midwifery care in four villages in Afghanistan covered by the project, so as to reveal challenges and improve services in rural and conflict-affected areas of the country.

**Methods:**

An exploratory case-study approach was adopted. Fourteen in-depth interviews and four focus-group discussions were conducted. A total of 39 women participated – 25 who had given birth during the last six months, 11 mothers-in-law and three community midwives in the provinces of Kunar and Laghman. Data generated by the interviews and observations was analysed using thematic content analysis.

**Findings:**

Many of the women greatly valued the trained midwives’ life-saving experience, skills and care, and the latter were important reasons for choosing to give birth in a clinic. Women further appreciated midwives’ promotion of immediate skin-to-skin contact and breastfeeding. However, some women experienced rudeness, discrimination and negligence on the part of the midwives. Moreover, relatives’ disapproval, shame and problems with transport and security were important obstacles to women giving birth in the clinics.

**Conclusions:**

Local recruitment and professional education of midwives as promoted by Afghan authorities and applied in the project seem successful in promoting utilisation and satisfaction with maternal and neonatal health services in rural Afghanistan. Nevertheless, the quality of the services is still lacking, with some women complaining of disrespectful care. There seems to be a need to focus more on communication issues during the education of midwives. An increased focus on in-service training and factors promoting quality care and respectful communication is necessary and should be prioritised.

## Background

After decades of war and conflict, the situation for the people of Afghanistan continues to be difficult. The country has one of the highest maternal mortality ratios (MMR) in the world (396/100,000 live births), with considerable variation between urban and rural areas – 253/100,000 and 620/100,000 respectively [1]. In fact, no more than 45.5% of Afghan women receive antenatal care and support from a professional health worker [2], and that percentage is decreasing, in part owing to a difficult security situation. In some provinces only 38.6% of women have access to a skilled birth attendant when in labour, which is about half of the world average of 70.5% [3]. This implies that seven out of ten women give birth attended by a traditional birth attendant or a relative – or alone. Women of a reproductive age (15 to 49 years) account for 22% of the population of Afghanistan [4], and the main challenges for childbearing women are access to skilled birth attendants, emergency obstetric care and reliable contraception. Such challenges to women’s health need a continuous focus [5, 6].

**Fig. 1:**
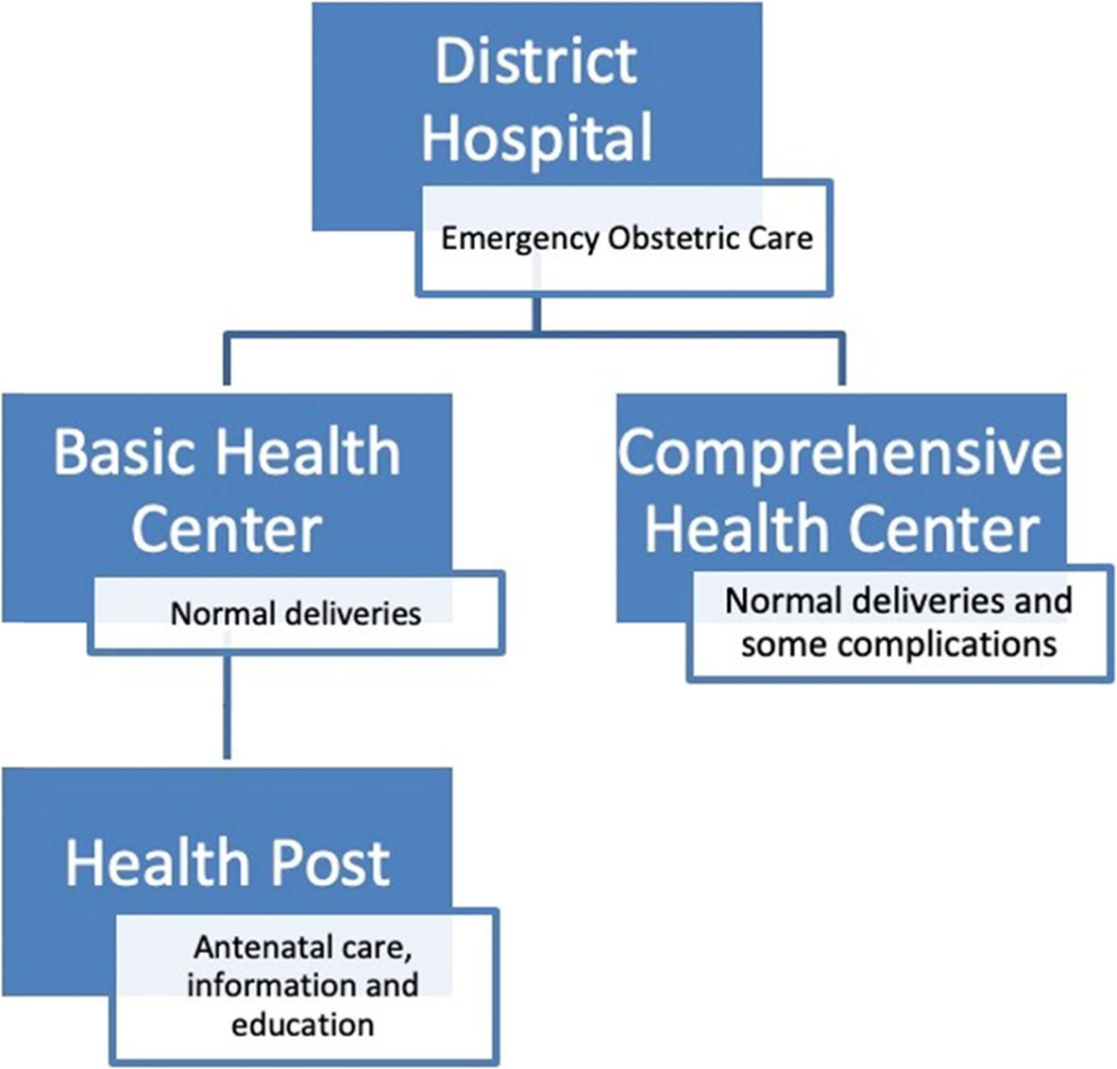
Health-care service at district level

The health sector in Afghanistan has three levels of service delivery: Health posts at community or village level; Basic and Comprehensive Health Centres (BCHC) in larger communities at district level; District, Provincial and Regional Hospitals providing secondary and tertiary services at provincial level [7] (Fig. 1).

Health posts offer antenatal care provided by community health workers, but no delivery care. Both Basic and Comprehensive Health Centres offer antenatal, intrapartum and postnatal care provided by midwives. A Comprehensive Health Centre offers services for a larger population than a Basic Health Centre, and also provides blood transfusions. A District Hospital should offer Emergency Obstetric Care including anaesthesia and surgery, e.g. caesarean section [7].

The Basic Package of Health Services (BPHS) was introduced in 2003, to address the country’s poor health indicators. The maternal mortality ratio in Afghanistan was by then 941/100,000 [1], and in some areas of the country 1600/100,000, which was among the highest number ever recorded [8]. After implementation of BPHS, both access to and utilisation of health-care services increased [8, 9].

Afghanistan has committed itself to the UN Sustainable Development Goals [10]. In order for the country to attain the goal of reducing maternal and neonatal morbidity and mortality, access to quality health care needs to be improved, and a focus on improving midwifery services is one of the important means of attaining these goals [10, 11].

### Education of midwives in Afghanistan

The profession of midwifery in Afghanistan was established early in the twentieth century, when 12 women from King Amanullah’s family were sent abroad to receive education as midwives. During the following years, midwives were educated and recognised as respected health-care providers. After the Soviet invasion of 1979, some girls got free nurse-midwife training in Russia and in towns such as Kabul and Jalalabad, as experienced by one of the authors (KS). Yet after the onset of civil war involving the Mujahidin and the Taliban, and particularly after the Taliban took control in 1996, women were denied schooling, and the country’s well-established health-care system was devastated [12]. This has had a lasting impact on Afghan women’s access to expert help during childbirth. In 2002 there were only 467 educated midwives nationwide, and their skills levels varied considerably. With international support, the Ministry of Public Health started revitalising the education of midwives in accordance with the standards of the International Confederation of Midwives (ICM) [5].

An educated, professional midwife is, according to The International Confederation of Midwives, ‘ … a person who has successfully completed a midwifery education programme that is based on the ICM Essential Competencies for Basic Midwifery Practice and the framework of the ICM Global Standards for Midwifery Education, and is recognised in the country where it is located; who has acquired the requisite qualifications to be registered and/or legally licensed to practice midwifery and use the title ‘midwife’; and who demonstrates competency in the practice of midwifery’ [13].

Since 2002 the Afghan government and the international community have focused on development of the midwifery profession through diploma-level educational programmes of various lengths [14]. Midwives are educated through two different routes for the primary and secondary health sectors. Hospital Midwifery Education (HME) builds on 12 years of schooling, whilst Community Midwifery Education (CME) requires a minimum of 10 years’ schooling and prepares midwives to work in rural Health Centres [14]. The programmes’ curricula are regulated by the national Afghan Midwifery and Nursing Accreditation Board [5]. In the CME programme local women are recommended for further education. The intention is for trainee midwives to go back to and work in their communities after graduation [14]. Following implementation of the CME programme, the number of women attended by a skilled birth attendant has increased by 28%, and a reduction in MMR has been observed [15]. More recently, Kabul University also started offering a four-year Bachelor of Midwifery [6].

The NGO Norwegian Afghanistan Committee (NAC) has supported the education of midwives in Afghanistan since 2002, and since 2009 has had the professional cooperation of The Norwegian Association of Midwives (DNJ). The NAC programme *Advancing Maternal and Newborn Health in Afghanistan* educates midwives and nurses at different levels, including through HME, CME and Community Health Nurse Education (CHNE). In the NAC-supported midwifery programmes, subjects such as domestic violence, anti-corruption and peace work are included in the curriculum. Currently NAC is running health-education programmes in six provinces in Afghanistan. The project is funded by The Norwegian Agency for Development Cooperation (Norad), and approximately 1000 midwives have graduated from the programme since 2002.

Although this project has been successful in terms of deploying midwives in rural communities, it is not clear how women value current access to midwifery care during pregnancy and childbirth. There is in any case little research into how such interventions have been received by Afghan women. Both the security situation and the geography – with difficult transport and lack of infrastructure – have limited such studies.

### Aim

The aim of this case study is to explore women’s experiences, perceptions and utilisation of local professional midwifery services at the time of pregnancy and childbirth in rural Afghanistan. It is intended to provide important insights into issues that need attention in order to further improve the education of workers in the field of reproductive health care and the organisation of services, and to increase women’s access to quality care at birth. In order to attain the Sustainable Development Goals of reducing maternal and neonatal morbidity and mortality, access to health care and barriers limiting it are particularly important for people in fragile and conflict settings and must be improved [16].

## Methods

### Study design

An explorative case-study approach was adopted for this research [17]. This type of study, involving different methods of data collection such as participatory observation, in-depth interviews and focus-group discussions (FGDs), can promote an in-depth understanding of events in their natural context, namely women’s experiences of midwifery services in rural Afghanistan. Elements of participatory research were also chosen, by collaborating with local stakeholders, through the Norwegian Afghanistan Committee in Kabul, as well as international stakeholders such as universities, the Norwegian Midwives Association (DNJ) and the International Confederation of Midwives (ICM). The primary investigator (TT) and designer of the study, a nurse/midwife with more than 30 years of clinical and tutoring experience and who has contributed in the Norwegian Afghanistan Committee’s education of midwives project since 2009, facilitated participation in the study. The aim of participatory research is to promote effective interventions that may redress power imbalances, facilitate mutual benefit among community and academic partners and promote reciprocal knowledge translation, incorporating community understanding into the research [18].

### Study setting and data collection

**Fig. 2:**
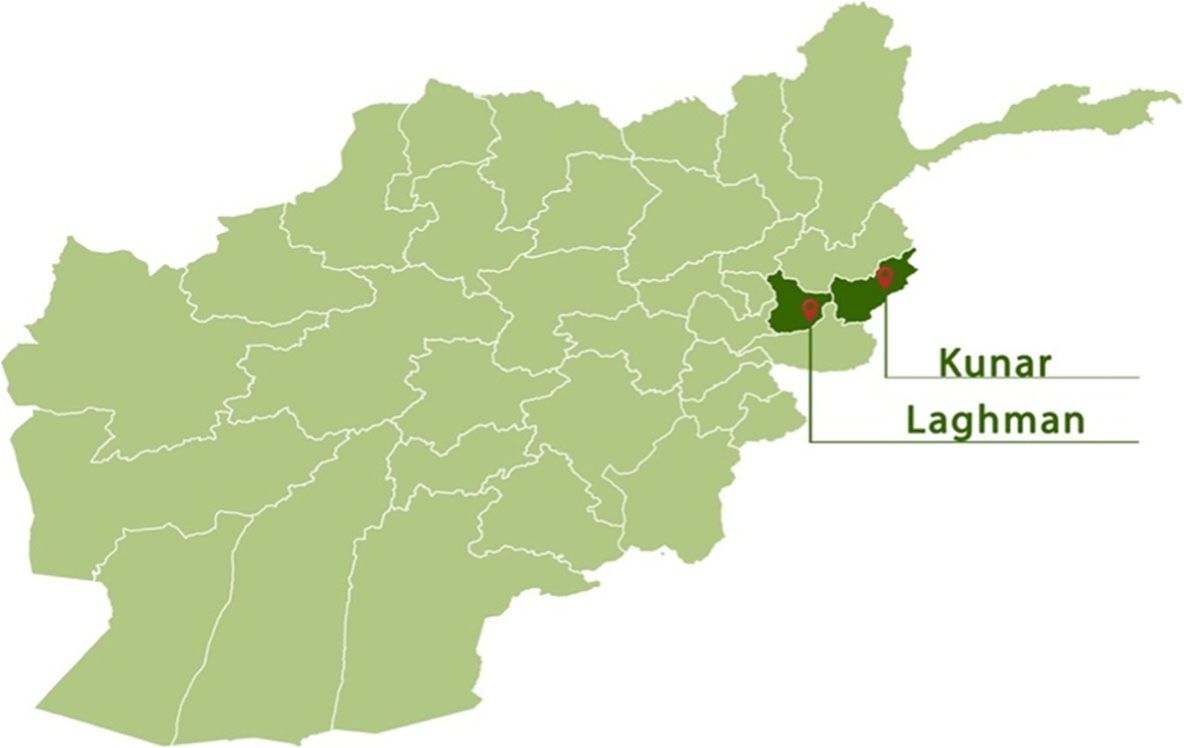
Study area; Kunar and Laghman provinces (Mustafa Savari, Norwegian Afghanistan Committee, Kabul)

The study was conducted in the provinces of Kunar and Laghman, two of the provinces where midwives educated through the NAC and DNJ programmes are working (Fig. 2).

Kunar is in the northeast of the country. The province is predominantly rural, most of the area being mountainous or semi-mountainous, and the geography makes movement difficult, necessitating movement on foot, as well as transportation using pack animals or motorised vehicles. The population is approximately 428,800, the majority belonging to the Pashtun ethnic group [19]. The major livelihoods of Kunar’s inhabitants include animal farming and forestry, with crop production practised along the Kunar river. The overall literacy rate (6+ years of age) fell from 32% in 2005 to 20% in 2011. Several schools have been closed down in recent years owing to increasing control by the so-called Islamic State (IS) and the Taliban (personal communication, AHW). Only 13% of women in Kunar are attended by a skilled birth attendant when giving birth (2011) [19].

The neighbouring province of Laghman is located further west, in the hilly areas of the Hindu Kush mountains, and has approximately 435,000 inhabitants. Here, too, the Pashtuns are the major ethnic group, other groups including the Tajik and the Pashai. The province is known for its lushness, and people make a living from farming fruit, vegetables and crops such as rice, wheat and cotton. The literacy rate in Laghman is currently 26%, representing an increase from 14% in 2005, and the percentage of births attended by a skilled birth attendant increased from 3% in 2005 to 36% in 2011 [20]. There are considerably more midwives in Laghman than in Kunar [21]. This may in part be because of the increased number of conflicts and the greater influence of the Taliban and IS in Kunar, as well as the shorter distance from Laghman to the midwifery educational facilities available in Kapisa province (personal communication, AHW).

### Study population and recruitment

A list of villages in the two provinces was developed, and four villages (two in Kunar and two in Laghman) were selected, based on what was feasible for the research team.

The two villages in Kunar are located close to the river, while the two communities in Laghman are close to the mountain. Most of the people living in these villages are very poor. Many of the houses, which are well kept and clean, are made from mud. They contain little furniture, and the beds are placed outside the houses and serve as both beds and chairs. Often, several houses share an open oven in the courtyard, where women meet for cooking, cleaning and socialing. The girls usually take care of their younger siblings. Many of the children are poorly dressed, wearing no pants or shoes, even though boys seem better dressed than girls. In the rocky Laghman villages, cultivation is difficult, and many men periodically leave their family to try to get a job in Pakistan or Iran. This means that many women, for long periods, live alone with their children, under the protection of older male family members.

A total of 39 women from these four villages participated in the research. Women who had given birth within the past 6 months, either at home, with help from another woman, or in a facility, were selected for the individual interviews. Mothers-in-law, women attending those in labour and childbirths in the community as well as other local women participated in the Focus Group Discussions (FGDs). Educated Community Midwives working in the same villages were also selected as informants (Table 1).

**Table 1:** Inclusion criteria for in-depth interviews/FGDs

Women who have given birth within the past 6 months	
Women who are residents in the village	
Women who are able and willing to participate	
Women who have given birth at home with help from a traditional birth attendant (TBA), mother, neighbour or other person	
Women who have given birth in a health facility	
These women’s mothers-in-law	
Community midwives working in the village and/or the Basic Health Centre	
Traditional birth attendants/local women attending those in labour and deliveries	

A total of 14 women participated in in-depth interviews, and four FGDs were conducted. Their community leaders recruited the 14 women who were interviewed individually. These informants included three community midwives. Apart from the midwives, all women participating in the research were unable to read and write. All informants participating in the interviews were given Afghan pseudonyms. The age of these women ranges from 17 to 30. Table 2 provides an overview of the women included in the research.

**Table 2:** Participants in the in-depth interviews – age range 17–30, walking distance to facility, gravida/para and number of children lost/alive

Pseudonym	Walking distance to facility	Gravida	Para	Miscarriage	Children alive	Children born at home	Children born in facility
Afrooz		8	8	0	7	7	1
Bahar	2 h	9	9	0	7	8	1
Camila	4 h	3	3	1	2	1	1
Delara	1 h	6	6		6		6
Farzana	30 min	3	3		2	2	1
Hadiah	1 h	4	2	2	2	1	1
Ilhaam	3 h	2	2		2	1	1
Jaleela	30 min	8	8		8	5	3
Khandan	1 h	6	5	1	5	3	2
Maheen	30 min	1	1	0	1		1
Nadia	20 min	7	5	2	5	4	1
Paksima (Midwife)		0	0				
Ramineh (Midwife)		3	3		3		3
Shabana (Midwife)		0	0				

### Data collection

The data from Kunar and Laghman were collected from July to September 2017 by Afghan research assistants, two from the NAC office in Kabul and two midwives from Laghman province. Fourteen interviews and four FGDs were conducted. The two interviewers from Kabul travelled to Kunar and stayed there for 2 weeks during the data-collection period. In addition to having detailed knowledge of Afghan culture, the interviewers closely observed the local context and traditions during their stay.

The four interviewers had previously participated in a qualitative-research-method course conducted by NAC during spring 2017. An interview guide was developed and was tested prior to data collection (see: Additional file 1).

The interviews were conducted in Dari/Pashto, the mother tongues of the informants and the interviewers. The continuous difficult security situation in Afghanistan in general and for women in particular made the data collection challenging, as none of the informants wanted to be recorded during the interviews. All the interviews were therefore conducted by the two data collectors working together, one of them taking notes and the other facilitating the conversations. The interviews were later translated from Dari (or Pashto) into English. The interviews and FGDs each lasted about an hour and took place in private houses and local Basic Health Centres.

### Conceptual framework

In 1978 Tanahashi described the AAAQ framework, which comprises the four underlying elements needed for attainment of the optimum standard of health care, including maternal and child health care. These elements are *Availability, Accessibility, Acceptability* and *Quality* [22]. *Availability* means the health facilities available at local level and to a sufficient extent, involving a sufficient number of health-care workers with the right experience and skills, as well as the essential drugs and equipment. *Accessibility* refers to physically accessible and affordable health-care services for everyone, particularly vulnerable groups. The services must meet people’s needs and be affordable, i.e. involve reasonable fees. The services must furthermore be *acceptable*, respectful of medical ethics and sensitive and appropriate to the different cultures and genders. Lastly, the health services must be of a good medical *quality*, and people must be treated with respect. A lack of quality of care in health services will have implications, particularly as regards the accessibility and acceptability of care [5, 22]. In the 2014 State of the World’s Midwifery report the AAAQ dimensions are seen as being both essential and important to attainment of an available, accessible and acceptable midwifery service of a good quality [5].

**Fig. 3:**
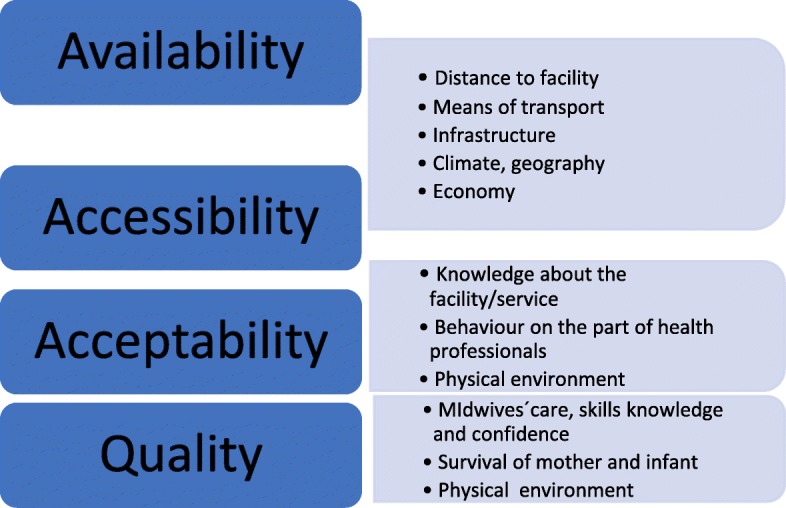
The AAAQ framework

In this study the AAAQ framework (Fig. 3) was applied, to facilitate understanding of how the NAC/DNJ intervention has modified factors that constrain women’s access to antenatal, intrapartum and postnatal care, as seen from the perspective of local users of services.

### Data analysis

**Fig. 4:**
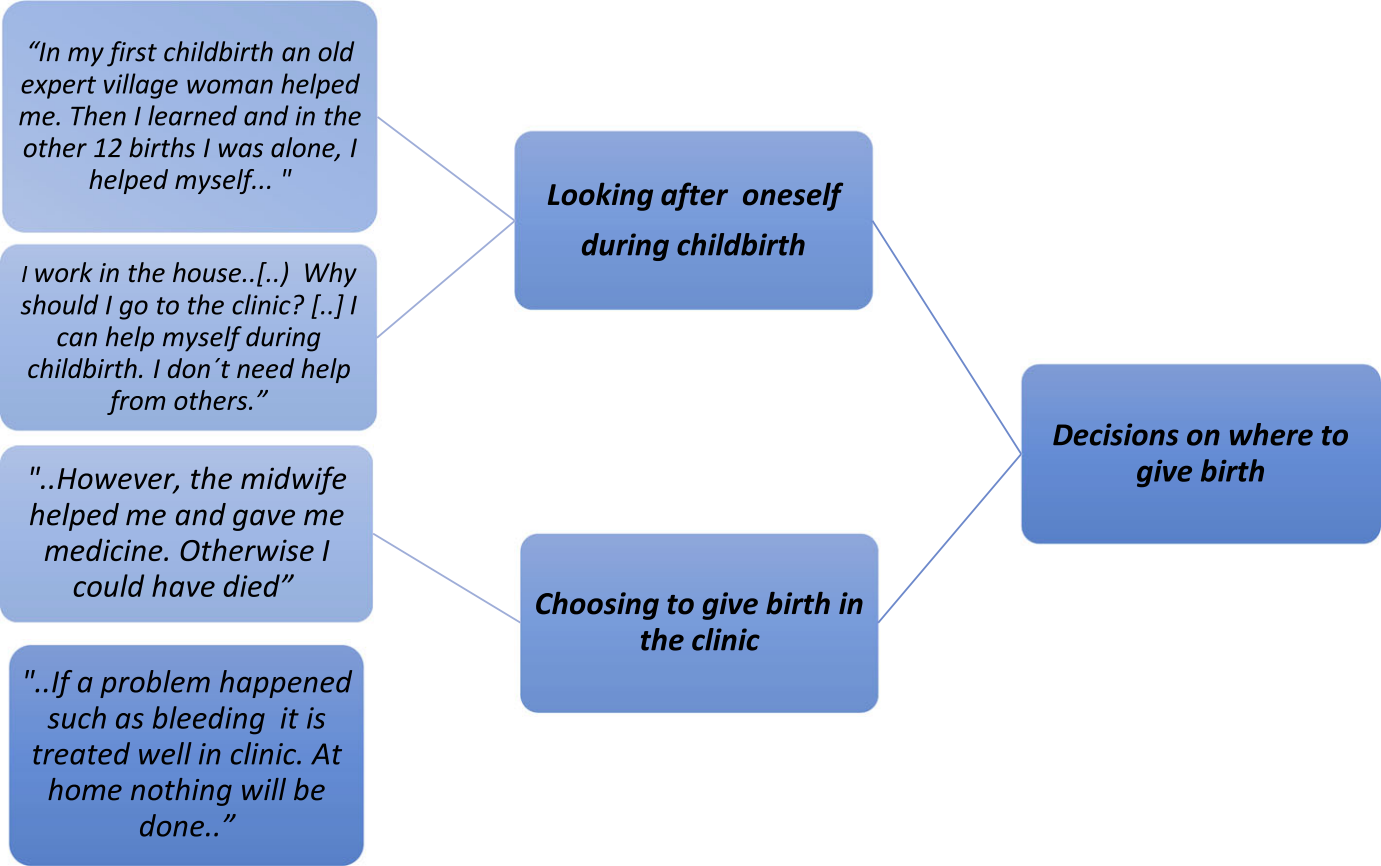
Examples from the thematic content-analysis process

The data was analysed using content analysis and thematic analysis, including a five-step procedure [23, 24]. Firstly, the field notes were read and translated into English by the data collectors in Afghanistan. Secondly, after reading and rereading the translated field notes, meaning units were identified and transformed into condensed units by the principal investigator (TT). Thirdly, the units were encoded, and topics and categories were labelled (Fig. 4). Fourthly, the number of units was reduced, and key units were identified, grouped and examined further. And lastly, the interrelationship between the units was examined.

### Findings

The findings are presented in accordance with the three major topics identified from the data: (1) Decisions on where to give birth, (2) Access to health facilities and (3) Receiving and evaluating midwifery care.

The 14 informants were all relatively young women, aged between 17 and 30, although most did not know their exact age. Despite their young ages, most of the women had previously been pregnant and given birth, some of them several times, and some had experienced losing a child. Most of the participants told the research team that they had given birth at home (Table 2).

### Decisions on where to give birth

Women in the study areas had access to midwifery care throughout pregnancy and childbirth, although for most of them the nearest health facility was over an hour’s walk away (see Table 2). Whilst most of the women were aware of the benefits of midwifery care, it turned out that not all of them used such a service. There were several reasons for this, and they will be discussed further. A number of women managed to look after themselves during labour and childbirth, and stated that they did not need or want midwifery care.

#### Looking after oneself: “I had 13 babies born at home”

Most of the older informants stated during the focus-group discussions that it is best to give birth at home, and that giving birth in a health facility is a modern practice they did not feel confident with. Some managed labour and childbirth by themselves:

*“I had 13 babies born at home. [ … ] In my first childbirth an old expert village woman helped me. Then I learned, and in the other 12 births I was alone, I helped myself … […] When a woman today becomes pregnant, she leaves her work and goes to the clinic … The women are so pampered … it’s like a diversion for them, they pass the time …”* (Woman, FGD 1)

A younger woman seemed to have a similar opinion:

*“If the woman does not feel pain, why should she go to the clinic? Some women just pass the time … Some women just waste time … I don’t like to idle away the hours with such unimportant issues … I work in the house, with the children and the animals. Why should I go to the clinic? […] I can help myself during childbirth. I don’t need help from others.”* (Afrooz)

However, during their pregnancies and after talking with other women, some women had changed their minds about where to give birth, especially after having trouble. One woman explained:

*“I had three children at home with a lot of problems. My neighbour advised me to go to the clinic. When I went there the midwives behaved so well and I was very happy and satisfied.”* (Khandan)

Thus some women, regardless of their age, felt confident about coping with childbirth on their own. As has been observed elsewhere in Afghanistan, some viewed pregnancy and childbirth as natural processes that should not require external help [8]. Such cultural attitudes may on the one hand reflect resilience, but on the other hand represent barriers to safe childbirth in the event of unexpected problems and emergencies.

Besides those women who claimed that they could manage to give birth on their own, some women expressed that they would have opted for the clinic but were not allowed to do so by their husbands or in-laws.

#### Choosing the clinic: depending on others’ approval

In Islam, pregnancy and childbirth are considered to be a special time of life, and religious traditions and rituals become more important to pregnant woman. An expectant mother prays and reads the *Qur’an* more often. As long as she is taken care of by female relatives, seeking professional health care during labour and childbirth may appear unnecessary [25]. Also, according to Islamic tradition Afghan women need permission from and accompaniment by a close male family member – a *Mahram* – in order to seek professional health care and to go to a health facility [25]. Frequently, husbands and in-laws did not consent to women giving birth in a clinic. However, during the discussions many of the research participants agreed that it is better to give birth in a clinic. Notwithstanding, one of the older women confirmed the general state of women’s lack of freedom to choose where to give birth: *“It is much better to give birth in the clinic than at home, but unfortunately most women are not allowed to go to the clinic …*” (Woman, FGD 2).

Other research participants explained that women should only use health facilities in emergency situations, the outcome sometimes being like what Nadia experienced:

*“I was pregnant in the fourth month and suffered from pain for four days. My in-laws didn’t agree that I should go to the clinic; they said it was shameful. Only when I became unconscious they took me to hospital. But I lost my child.”* (Nadia)

In-laws’ opinions seemed to have a powerful influence on the women: *“My in-laws opposed me about going to the health facility. They felt ashamed and said it was not good for a woman to go to the clinic.”* (Khandan).

One woman explained that her first pregnancy ended in a miscarriage. She was bleeding severely, but her mother-in-law still did not want her to go to a clinic. When she was in shock and unconscious, they finally carried her to the clinic, 4 h away from their home. “*The midwife said to us: you came very late to the clinic ...*” (Camila).

As illustrated above, some families considered that attending a clinic for childbirth was shameful. In fact, some women expressed personal shame about having to reveal their bodies, and these women did not want to give birth in health facilities.

#### The clinic as a site of shame

The privacy and intimacy of labour and childbirth apparently influence the choice of place of birth, and many of the women had decided to give birth at home because they felt it was disgraceful to expose themselves to a health professional. Some felt this to be so intimidating and embarrassing that they themselves refused to go to a facility when in labour:

*“They [the family] wanted to help me when it was time for the baby to come. They wanted me to go to the clinic. I refused. I don’t like women to see me … there … during the birth of the baby. I am ashamed during childbirth … So, I did it alone.”* (Afrooz)

Several women confirmed this with similar statements about a lack of privacy on clinic premises and the potential of shameful exposure to other women: “*The windows in the delivery room do not have curtains. The clinic has few beds … one woman was delivering on the floor with other women present. We don’t feel relaxed there …*” (Bahar).

Thus it seems that women face many obstacles and considerations when it comes to choosing a place for birth, owing to a combination of individual, cultural, religious and practical considerations. In a study in which Afghan women were asked about their customs and traditions during the perinatal period, the women emphasised that being a good Muslim is particularly important during this period, when, for example, undressing in front of a stranger and exposing oneself to a provider are seen as being inappropriate [25]. This interpretation might be particular to local cultural contexts in Afghanistan, as according to Islam a male doctor is also considered Mahram for women, meaning that he is allowed to see, touch and examine them for therapeutic purposes (personal communication by KS). Moreover, many women in our study also saw the benefit of giving birth in clinics, where they could get help in the event of complications. Apart from sociocultural barriers, as described above, infrastructural and environmental factors put constraints on women’s use of skilled care, as discussed below.

### Access to the health facilities

The villages included in the study are situated in relatively remote areas of Kunar and Laghman provinces, and the walking distances to the nearest Basic Health Centres varied from 20 min to 4 h. In the summer it is usually hot, and people seek shade from the sun. During fieldwork, for instance, discussions had to take place in shady areas between people’s houses. In wintertime there is often a lot of snow in these areas, making transportation difficult. The only routes into most of these villages are by roads that in most cases can only can be accessed by donkey or bicycle. When discussing transport, the women explained that very few people in their areas have access to a car. Some car owners would be willing to lend their cars out, but suspicion and security issues affected their trust and willingness to do so. Some families had a donkey they could use for transportation.

#### Distance and the need for transportation

Most of the women in the study lived far away from a health facility (see Table 2). Some women did not give birth in clinics because of the distance from their homes. This could have fatal consequences:

*“Too often I gave birth to a dead baby ... [...] When I was full-term pregnant my labour pains did not start. In all my pregnancies my labour pain did not start ... So, I went to the clinic ... The clinic is two hours’ walk away, far from my home. When I came to the clinic the midwife gave me an injection to start the labour, but the baby was already dead in my womb ... The midwife advised me to come [early] to the clinic the next time. If not, my baby might die, again.”* (Bahar)

Families and communities would often help the women to reach a facility, when judged necessary. One woman who lived 1 h’s walk from the clinic explained that it was hard to get there, but each time she was brought to the facility by her spouse. “*My first five children were born in the clinic. My husband carried me to the clinic.*” (Delara).

Parents or other villagers would help as well: *“My father’s family and the village people prepared transportation for me,*” Afrooz said.

Thus in spite of the problems of distance and poor means of transportation, it seems that a number of families and women tried to find solutions to the transport issue, as well as the costs.

#### Coping with financial constraints

The people in the villages represented in the research were generally very poor, and during the FGDs poverty was often mentioned as a general problem that prevented them from having access to transport. Nevertheless, it seemed that many women and their families were trying to plan transport when their labour started. Many women explained about various efforts on the part of relatives to facilitate transport in case of need.

*“The clinic is one hour by foot from my house. I had planned to give birth at home, because we did not have money for transport. But my husband took the decision and borrowed money so I could go to the clinic [ … ] If you have money it is good to give birth in the clinic. But you need money for transportation and medicine.”* (Delara)

Some women explicitly planned to give birth in the clinic, and prepared themselves as best they could: “*I got advice from the midwife to give birth in the clinic. We are poor ... So, when I was pregnant, I sold a sheep and saved money for the birth of my baby.*” (Afrooz).

In spite of being prepared for the financial costs of safe birth care, it is difficult for most women and their communities to prepare for the lack of security during travel to clinics.

#### Going for safe births on unsafe roads

The insecurity situation in the study area, particularly at night, posed a risk to women who wanted and needed to go to the clinic, as explained by a community midwife: “*Every mother should have antenatal care. However, many women don’t come because of the poor security situation.”* (Paksima, midwife).

Another midwife specified what people were afraid of:

*“The most challenging thing is safety. This problem with lack of security is very, very difficult and challenging for us, both for me as a midwife to go to work, and also for the women in labour. When going to the clinic we use the same road as the bombers … we are on the roads where something happens every day … Something ... an explosion, a suicide … or something else … It makes moving from one place to another very dangerous and difficult.”* (Ramineh, midwife)

The ongoing conflict and insecurity situation in Afghanistan is well known, is currently worsening and is an international concern. This pervasive situation reduces the availability of healthcare and limits access to essential health-care services in the long run [8, 26]. This has a secondary impact on health-care workers and health-care services, as they have become integrated into the conflict. Nevertheless, health-care providers continue to provide services and expose themselves to this insecurity [8, 26].

### Receiving and evaluating midwifery care

The women had different views and experiences of the midwives and midwifery services. Some women had given birth both at home and in a clinic, and were able to compare the experiences. Some had experienced complicated deliveries at home as well as in the clinic, and this resulted in both positive and negative evaluations of expected and received care.

#### The good experiences of midwives: “… they are like members of the family”

Most women expressed that the midwives in the clinics were knowledgeable, skilled and confident, and that they cared for the women patients. “*When we come to the clinic the midwives treat us well during labour and childbirth. We feel that they are not like other health professionals ... they are like members of our family!*” (Woman, FGD 4).

Although some were initially unsure and sceptical about midwifery services, most women claimed to be satisfied after delivering in the clinics, and after experiencing the midwives’ vital professional expertise: “*After the delivery I had very serious bleeding. I was very afraid and worried. However, the midwife helped me and gave me medicine. Otherwise I could have died.”* (Khandan).

Women expressed genuine gratitude:

*“I am really thankful to the midwives, they are really nice and patient, may Allah keep them happy [...]. It is very important and good to give birth in the clinic rather than at home. If a problem happens such as bleeding or the woman collapses, it is treated well in the clinic. At home nothing will be done.”* (Maheen)

The fact that the midwives explained about what was happening and why created trust among the women:

*“My last baby was born in the clinic. The midwife assisted me, she was kind and friendly. I had severe bleeding. The midwife said that a few pieces of placenta were left in the womb. She helped me, took the pieces out and stopped the bleeding.”* (Bahar)

Moreover, the mothers seemed to appreciate the way their newborns were put in close bodily contact with them after delivery, as well as the prompt initiation of breastfeeding. “*The midwife was so kind. She put my baby on my chest after he came out.*” (Afrooz).

Another woman explained: *“The midwife put my baby on my belly. After two hours I started breastfeeding.”* (Bahar).

The midwives in the village clinics were taught and pursued “Baby-friendly environment” guidelines – a programme launched by the WHO in 1991 with the aim of improving maternal health care. This includes, for example, immediate skin-to-skin contact and initiation of breastfeeding immediately after birth. These practices seemed to be particularly appreciated by the women, even though there is no tradition of immediate skin-to skin-contact and early breastfeeding in Afghanistan [26, 27].

#### Negative experiences of midwifery care: “… she called me a donkey”

In spite of the many positive experiences in the various communities, a few women shared their negative experiences regarding the midwives and the care they received.

*“My last childbirth was in the clinic ... The midwife assisted me, but she was not a kind woman. I did not feel comfortable. I had bleeding, and she had to send me to the hospital. Giving birth at home is risky … The clinic is better ... However, the midwife was not kind. I did not feel good.”* (Farzana)

Some women even felt harassed and intimidated:

“*I had severe pain, I was crying, I was moving around, I couldn’t be calm. The midwife became angry and said “You behave like a donkey, you are not a human being!” [ … ] Sometimes midwives become so angry with mothers, I don’t know why. [...] I am happy that she helped me, but I feel so sad because she called me a donkey.”* (Camila)

Other women explained that the physical environment and conditions in the clinic were additional reasons for not giving birth there. They complained about the poor equipment and the staff’s behaviour.

*“I went to the clinic to give birth. In our clinic there are midwives, but no female doctor [...] There was no light or fan … They did not turn on the generator ... it was so hot! [...] The midwife examined me, and afterwards she went to sleep. When I called for her, she became angry and did not behave well [...] They don’t pay attention to the patients.”* (Woman, FGD 2)

In order to understand such poor caring behaviours in midwives, it is important to mention the professionals’ workload, involving a poor shift system for the midwives. Sometimes they had to work for 24 or 32 h consecutively. Moreover, it is suggested that some midwives suffer domestic violence in their family because of their work, and this may also result in disrespectful care of their patients (personal communication by KS).

In spite of these negative experiences, women expressed that midwifery services were appreciated by many, and that midwifery seemed to be an increasingly valued career path for women in the communities.

#### Becoming a midwife: a valued professional career

The discussion during the FGDs and interviews usually began with talking about the trained midwives operating in the localities. In some of the villages the midwife was the only educated health-care provider in the community, and the profession as such represented a rare opportunity for education and increased status for women. This was discussed during some of the conversations.

*“I am really enthusiastic about this education, and I wish I could be a midwife in order to help my family and the villagers. But unfortunately I got engaged during my school period, and after marriage my husband didn’t want me to continue my schooling, he didn’t understand the value of education.”* (Woman, FGD 3)

Some older women expressed how the value of education had changed over time, as had their own ideas about it.

*“Before, we did not allow our children to learn and to get an education, I wanted my children to work in the field instead. Now I know and understand and meet educated people, I see their attitude and value in society. I want my granddaughters to learn and get an education.”* (Woman, FGD 2)

Another woman said something similar, and intended to involve her husband in promoting their daughter’s education:

*“Some people agree about women getting an education. I want my daughter to learn and get an education. I want to ask my husband to allow her to start [on the midwifery programme], if not I will make him allow her.”* (Woman, FGD 3)

Some even stated the importance of the profession at national level: *“I want my granddaughter to learn and to get [midwifery] education, to serve the people of Afghanistan.”* (Woman, FGD 3).

Thus training in midwifery seemed to provide some status and recognition, and was apparently considered to be an important pathway in terms of increasing education for women in the country.

## Discussion

The aim of this case study was to explore women’s experiences, perceptions and utilisation of local professional midwifery services at the time of pregnancy and childbirth in rural Afghanistan. Afghan women’s utilisation and experience of health-care services during pregnancy and childbirth are affected by several factors, such as accessibility of services and lack of transport, particularly in rural areas of the country. In addition, financial and cultural barriers, women’s level of education, their knowledge of danger signs and poor health-care provision are important factors promoting non-utilisation of professional health services [28–30].

Women’s experiences and utilisation of services in the four villages represented in the research, in Kunar and Laghman provinces, were predominantly positive. Most of the women were content with the services received, and most of them did attend the recommended antenatal care, and apparently appreciated the value of a skilled midwife in terms of health and survival for mothers and infants. Many told stories of serious consequences when not attended by a professional midwife, or when referred in time to a facility with emergency obstetric care.

However, women’s utilisation of midwifery services was still influenced by sociocultural values and attitudes that were not always favourable for women seeking skilled care. Value and attitudinal barriers included a lack of permission from relatives/in-laws, in addition to women’s own, sometimes negative, perceptions of midwifery care. Women also felt shame about exposing themselves to others during birth, and for this reason some did not use midwifery services. Others were not satisfied with the service and care that midwives provided. Despite these experiences and barriers, most of the women interviewed in our study preferred to give birth in a health facility with help from a midwife, and did so. This indicates that the national midwifery education programmes, such as the NAC project, which focuses on recruiting women from the community into midwifery, have had a positive effect on women’s access to midwifery services.

### Availability of and access to midwifery services

Through the programme *Advancing Maternal and Newborn Health in Afghanistan*, community midwives are educated and deployed in rural parts of Afghanistan. Women’s access to health-care services in rural parts of Afghanistan is still limited. In the areas where this study was conducted, women generally do have access to midwifery health care during pregnancy and childbirth [21]. As shown in our study, despite midwifery services being available locally there are still many barriers that restrict women’s access to and use of these services, such as transport, security and financial problems. A literature review from 2016 confirms these barriers to access to health-care services in Afghanistan, and mentions geography and gender issues as key factors [8]. One must also consider the fact that professional midwifery does not have a long history and tradition in Afghanistan, and women’s experience of professional birth attendants is limited [12].

All the women in our study were familiar with the midwifery services. The fact that the midwives were known women recruited from their own communities could contribute momentum in terms of increasing awareness and furthering utilisation of midwifery services [6]. In a study from eight different provinces of Afghanistan (2013), many midwives felt supported by their own communities and believed their presence in these communities would raise awareness of the benefits of midwifery services [14].

Several studies show that awareness and knowledge of midwifery services increase the utilisation of such services during labour and childbirth. Our findings are consistent with these studies. A cross-sectional study from Ethiopia showed that raised awareness and inclusion of both husbands and other family members in maternal health care seem to have increased the use of maternal health-care services [31]. A study from Nepal described the consequences of the opposite situation, namely how women’s insufficient knowledge and awareness of the importance of a skilled birth attendant restricted their utilisation of the services [32].

Despite the fact that the women in our study had access to a health facility, some of them still preferred to give birth at home, claiming that they could manage labour and delivery alone, without help from others. There may be many reasons for this, such as experience-based self-confidence in handling a childbirth alone, as well as families’ opinions and women’s shame in exposing themselves to strangers, as expressed by a number of women interviewed during the research. In fact, shame may also be associated with the exposure of loss of blood and bodily fluids during birth. During a NAC meeting in the province of Kapisa, several women told the team that they preferred to give birth at home, alone. A woman explained: “*When my husband comes home, the baby is born and I have washed and cleaned up everything*”, thus expressing the importance of removing birth dirt, this “matter out of place” which according to Mary Douglas often symbolizes danger and power [33].

A cross-sectional study from all 33 provinces of Afghanistan shows that older women use a skilled birth attendant less often than younger ones do, and that factors such as distance to facilities and availability of female health-care providers are important determining factors in this decision [28]. Other reasons may be that older women have most likely given birth before, and if these births were uncomplicated they chose to give birth at home again the next time. Older women may also hesitate to use midwifery services because they are more faithful to traditional beliefs around pregnancy and childbirth [8, 27]. Mayhew et al.’s cross-sectional study from 2008 showed that women aged 30–39 used skilled birth attendants less than did younger women, though these figures were not statistically significant [28]. In our study, younger women seemed to have more knowledge of midwives’ skills and the benefits of giving birth in the clinic, and they did their best to overcome barriers to accessing midwifery services. The women interviewed discussed the benefits of giving birth in health facilities with their husbands and relatives, and planned and saved money for transportation to such facilities. This may indicate changing norms and positive attitudes towards skilled birth attendance across gender and generations in local communities. A systematic review from nine low- and middle-income countries confirms that this trend towards change is occurring in several places, whilst illustrating the benefits and importance of men being increasingly involved in maternity care, with positive outcomes for the mother, the neonate and the family [34].

### Practical access to services

Geography and climate in Afghanistan are factors that clearly have an impact on utilisation of health-care services in general and maternal health care in particular. Long winters, often with a lot of snow, as well as a poor infrastructure limit people’s ability to travel. It can be difficult to get out of villages, whether on foot or on a donkey. In many villages few, if any, people have access to a car, and driving conditions can be difficult or impossible for long periods of the winter, and when the snow is melting in the spring.

The women in our study generally described both the midwifery services and health facilities as being accessible, even though most of them did not live close to health centres. Despite this, the women explained that long distances, difficulties with means of transport and security problems were obstacles to their using these services. Although the intention of the BPHS is to ensure access to health services for people in rural areas, these services are not accessible to everyone. Walking to a health facility with painful contractions can be a challenge for any woman, even when the distance is short. Mayhew et al. showed that in Afghanistan utilisation of health facilities for birth is related to the walking distance to the clinic – the greater the distance the less the use of a health facility [28]. Yet it seems that nowadays, 10 years later, women are more aware of the need to prepare for and cope with transport expenses, so as to ensure a safer delivery with skilled birth attendants, as shown in our study.

After 40 years of war, internal conflicts as well as the presence of the Taliban and IS make the security situation for people in Afghanistan extremely difficult [35]. The midwives in our study told us how women failed to use perinatal care services because of security problems, particularly at night. Nevertheless, most of the women wanted to go to the clinics, many of them accompanied by the local midwife. A systematic literature review from 2017 [36] showed that obstacles in terms of availability, accessibility and quality of care are particularly common in countries experiencing war and conflict, such as Afghanistan, which may be one of the countries most adversely affected by war and conflict anywhere in the world. Health indicators in general and with regard to maternal and child health in particular are mostly poor in such fragile environments, with limited access to both basic and emergency obstetric health care. Afghanistan alone represents 2% of neonatal mortality globally. Thus this conflict-affected country, as others, needs additional attention and resources, particularly when it comes to maternal and child health [36].

### Acceptability of the service

In Afghanistan’s sociocultural and religious context, pregnancy and childbirth are seen as particularly delicate and private issues, and health services aimed at women and children must accordingly be sensitive [8]. In many parts of the country it is culturally unacceptable for a female patient to be seen by a male health professional, and female patients typically need to be accompanied by a male relative [8, 25]. Some women in our study described such a lack of trust in and acceptance of public health services, thus showing the need to better integrate cultural sensitivity and respect for privacy and intimacy into maternity health professionals’ education.

In a war-afflicted society like that of Afghanistan, confidence in health workers as well as health workers’ own safety are especially important. The way midwives are recruited to the NAC project seems to have informed the local communities and made them conscious of the importance of a professional service, and of the midwife’s knowledge and skills and the difference they make for mothers and infants. However, some communities may in general have insufficient knowledge of midwives’ life-saving skills. Some women expressed having had a brutal experience of midwifery services, or had heard about other women who had lost their lives and/or babies in clinics.

A systematic literature review from 2014 describes barriers to childbirth in health facilities in 17 low- and middle-income countries. These barriers are similar to those found in our study, e.g. local traditions and influence of the family, availability, access and quality of care. Women in our study talked about family members who forbade them to give birth in a facility because this is not the tradition and it is perceived as being shameful. They talked about long distances to the facility and a lack of both money and means of transport. Many women in our study also experienced a lack of respect, privacy and intimacy in the clinic, and therefore decided to give birth at home. Families with social connections to skilled providers may be more accepting of the biomedical approach to maternity care and thus more willing to seek a facility-based delivery [37]. This highlights how family members’ opinions and decisions in some cases limit women’s use of midwifery services, and shows how these barriers can have fatal consequences for women. A study conducted in a suburb of Kabul back in 1996 shows that even in an urban setting most women preferred to deliver at home, only using birth in a health facility when expecting a complicated delivery, or in emergency situations. However, some women in this study highlighted security and cleanliness as reasons for giving birth in a health facility [38]. Lack of hygiene and cleanliness were also pointed out in our study as negative aspects of giving birth at a facility, along with the fear of being exposed to others. Nevertheless, in our rural study most of the informants seemed aware of the benefits of giving birth in a clinic, and this may illustrate similar health awareness and promotion potential in both rural and urban settings.

A study from 36 low- and middle- income countries, including Afghanistan [39], describes barriers, challenges and solutions to availability, accessibility, acceptability and quality (AAAQ) of maternal and neonatal health services. In the review the dimensions Acceptability and Quality are interrelated, since both acceptable and high-quality care require respectful, ethical and culturally appropriate attention on the part of health-care providers, e.g. respect for privacy and intimacy [39]. We apply the same approach, and this brings us to the perceived quality of services in our research locations.

### Quality of services

#### Appreciating life-saving care

Most of the women in our study appreciated the quality of the midwifery services, as regards both the professional and the interpersonal care they received. They expressed that the midwives’ expertise, in particular in emergency situations, their resultant ability to save lives and their care and kindness combined to create important reasons why they decided to give birth in clinics. This stance was supported by the women who were initially sceptical of birth in a health facility. A study from Ethiopia shows that women value giving birth in a clinic, since this includes medical interventions and the ability to deal with complications [40]. Also, in a study from Nigeria women expressed that the availability of knowledgeable and caring staff who could handle complications were important reasons for giving birth in clinics [41].

… *and care promoting early closeness between mother and neonate.*

A study from five different provinces in Afghanistan representing the country’s major ethnic, religious and language groups showed that a common tradition is for the baby to be given a bath immediately after birth, and for the mother to have a bath after 3 days. In this tradition a woman should not start breastfeeding before her breasts are clean, thus delaying the first breastfeeding by 3 days [27]. Another study confirms this custom, and describes a widespread Afghan practice whereby the neonate is fed with dates and honey before breastfeeding starts [25]. The fact that the baby is not put to the breast immediately may affect the success of breastfeeding, and may turn out to be an unfortunate norm when it comes to preventing malnutrition and optimising the child’s development [42]. In our study the women highlighted the fact that the midwives promoted immediate skin-to-skin contact and early breastfeeding as being particularly positive per se, thus demonstrating successful, empowering and health-promoting communication between the women and the skilled midwives.

#### The advantage of a familiar professional midwife

In cases such as in our study, where midwives are recruited from the local environment, and among local women, this might increase women’s and their families’ experience of safety and security. Midwives who operate within communities are furthermore able to provide a continuum of care through pregnancy and childbirth, as well as during the postnatal period, which is known to improve the utilisation of such services [43]. In fact, as observed in the villages included in the study, midwives in many communities of Afghanistan are the only professional health-care providers.

#### Delivery and lack of privacy

However, other women in our study were not satisfied with the care and treatment they received, and preferred to give birth at home even though they knew this could be risky. They were reserved and excessively shy when it came to exposing themselves to someone outside their intimate family circle, even in medically related contexts. They complained of lack of privacy and poor facilities in the clinic, such as lack of beds and curtains, and said that modesty and the shame of exposing themselves prevented them from giving birth in clinics. Lack of privacy during childbirth is described in two different studies from Nepal, in which women expressed the view that shame and shyness about showing their genitals were the main reason for not delivering in a health facility [32, 44]. These factors were perceived as being so important that even women who lived close to clinics often chose to give birth at home [32]. A study from Bolivia describes similar embarrassment about giving birth in a clinic, where women would “… be looked at” [45].

In our study, a lack of delivery beds meant that some women had to give birth on the floor, adding to their discomfort. A study from various maternity facilities in Kabul [38] showed that urban women also experience a lack of intimacy and even fear in clinics with limited resources in terms of beds and rooms. In fact, in overcrowded urban facilities lack of furniture and equipment may be even more challenging. According to a report from 2016 on maternal and neonatal care in the country, only 54% of facilities offered visual and auditory privacy in antenatal consultation rooms, and only 58% of facilities provided visual and auditory privacy in the delivery room [46]. Given the importance of promoting facility-based delivery in Afghanistan, the provision of basic and relatively cheap equipment ensuring a minimum level of comfort, privacy and dignity for women should be improved, thereby increasing the acceptability of services.

#### Disrespectful care of delivering women

Some women experienced disrespect on the part of the midwives, involving neglect and verbal harassment, and stated that the midwives did not provide good care. Disrespectful care and even abuse during labour and childbirth are documented in various studies. Women in Afghanistan frequently report disrespectful care, lack of compassion and abuse on the part of health-care providers [29], e.g. instances of women being shouted at, insulted or threatened during consultations [46]. Abusive behaviour during pregnancy and childbirth seems to be a widespread though underreported problem. A systematic literature review states that it occurs in many countries in Africa, Asia and the Pacific. Out of 81 included studies, 58 only mentioned negative attitudes on the part of providers [47]. In our study most complaints related to midwives’ attitudes – when they were perceived as lacking compassion for and interest in the individual woman. Kaartinen’s study from Kabul, Afghanistan (1996) confirms the fact that dissatisfaction with health providers’ behaviour is an old problem, and that health professionals’ skills, empathy and justice have long been important factors when deciding whether to give birth in a facility [38]. Neglect, lack of empathy, poor communication and even harassment during childbirth are also described in a study from Ethiopia [40], and a study from Kenya documents that health-care providers in facilities are perceived as being uncaring and discriminatory towards women in labour because of their low economic status [48]. Research in two hospitals in Tanzania [49], where observation of health-care providers’ behaviour was compared with women’s experience of disrespectful care and abuse, showed a significant higher prevalence of disrespectful care and abuse than the women themselves reported. This suggests that disrespect and abuse can be internalised and normalised by women in labour as well as by the health-care providers, and furthermore that some women might avoid reporting disrespectful care and abuse for fear of reprisal [49]. This could also be true for our study, suggesting that only a minority of the research participants would have complained of negative experiences. As explained in other settings, the asymmetric distribution of power between poor, illiterate women and the more educated health-care providers may create so-called norms of passivity, with the women not verbalising their negative perceptions [50].

However, the challenges facing professional health workers and how this may influence their ability to provide care need attention. As suggested in a report about maternal care in Afghanistan, gender norms and increasing insecurity may be factors underlying health workers’ performance [46]. For example, 20% of skilled birth attendants interviewed reported feeling that their own family responsibilities interfered with their work responsibilities, and 33% reported a lack of transport or safety as affecting their ability to do their jobs. Nearly a third also reported having experienced verbal, physical or sexual abuse at some point in their lives, which may have a lasting impact on physical and emotional wellbeing and/or the ability to care for others [46]. Moreover, the overburdened working conditions for some professionals, the lack of resources such as drugs and equipment and the potential lack of professional support some midwives may experience are factors that may suggest burnout, resulting in a lack of empathy and disrespectful behaviour towards their patients [5, 9]. The implications of mistreatment, and the fact that global evidence shows that fear of abuse in maternity care is a more powerful constraint on use of skilled care than barriers such as cost or distance [46, 51], confirm the importance of understanding how empathic and respectful communication and birth assistance can be reinforced.

Nevertheless, in our study women’s overall perception and experience of midwives and services were positive, and for most women the advantages seemed to outweigh obstacles such as long distances, transport difficulties and security problems.

## Strengths and limitations of the study

This study provided recent and original qualitative data material in a context in which it is difficult to do research for security reasons and owing to remoteness. Triangulation of three methods of data collection – in-depth interviews, FGDs and observation as applied by the local data collectors and the main researcher over time – strengthened the validity of the findings.

The interviews and discussions were run by four Afghan women and conducted in Dari/Pashto. These women are very knowledgeable about the local culture, traditions and rituals around the time of childbirth, in addition to having a deep understanding of the project and the training of community midwives. The joint critical analysis of the data by the local data collectors and the principal investigator (TT) is considered to be a strength of the research.

Owing to the difficult security situation in Afghanistan in general, and for foreigners in particular, it was impossible for the main researcher (TT) to run the interviews herself. Use of local research assistants with limited experience of qualitative research was a major challenge. There is a risk that women selected for the study were more in favour of the programme than others, and that as a matter of courtesy the informants emphasised the positive aspects of the midwifery project’s interventions. The data collectors might have been biased in their choices of interview respondents and interviewing processes, creating a more positive picture than warranted, since they all have a link to the NAC, either as employees or by virtue of having been educated through the NAC programme. In the process of translation from Dari/Pashto into English, important details might have been lost. Finally, the female data collectors had to overcome challenges such as transport, security and a limited time frame, which may have affected the data-collection process. The research team have been aware of and have critically discussed possible weaknesses, but we believe that the female research assistants’ background and skills were significant in making the study possible and relevant.

## Conclusion

The burden of maternal and child ill health in Afghanistan is one of the highest in the world, and it is being worsened by a long war. Our study explored women’s experiences in two rural provinces, where a midwifery-education project recruiting local women to the profession has been running, in line with the national midwifery-education programme. Our findings suggest that the midwife often becomes a central figure in local society, leading more women to choose to give birth in a health facility, when available and accessible. Most informants stated that the service was relatively satisfactory, and increased awareness of the importance of skilled care during pregnancy and childbirth was voiced by women, their relatives and the communities as a whole.

Nevertheless, issues of privacy and shame as well as the experience of disrespectful care affected the acceptability of midwifery services for some. An increased focus on respectful care and attitudes, and on communication in both pre-service and in-service training of midwives, is necessary in order to improve the quality of services. As regards research, it would seem important that one understand how to better prepare and support midwives operating in such demanding working conditions, and in this way strengthen the quality of care.

## Supplementary information

**Additional file 1.** Interview guide: In-depth interviews for women.

## Data Availability

The data set (anonymised transcripts of the interviews) used in the present study is available on request from: Centre Director, Centre for International Health University of Bergen Postbox 7804, N-5020 Bergen, Norway. Email: post@igs.uib.no

## Abbreviations

BPHS: Basic package of health services
CME: Community midwife education
DNJ: The norwegian association of midwives
FGD: Focus group discussion
HME: Hospital midwife education
ICM: International confederation of midwives
IS: The so-called islamic state
MMR: Maternal mortality ratio
NAC: Norwegian Afghanistan committee
Norad: The norwegian agency for development cooperation
TBA: Traditional birth attendant
