# Supplementary material for: “The midwife helped me ... otherwise I could have died”: women’s experience of professional midwifery services in rural Afghanistan - a qualitative study in the provinces Kunar and Laghman
Source: BMC Pregnancy Childbirth. 2020 Mar 6;20:140. doi: 10.1186/s12884-020-2818-1 (PMC7059669; doi:10.1186/s12884-020-2818-1)
Supplement: Supplementary file 1 — Additional file 1. Interview guide: In-depth interviews for women. [file 12884_2020_2818_MOESM1_ESM.pdf]

Interview guide: In-depth interviews for women

Do you have any questions about the study or the interview? If you agree to take part in the interview and are happy for us to record the conversation please state that now. I will make a note of this sheet to show I have witnessed your oral consent. [NB: if anyone else is present at the interview please check their consent and record who has participated in the interview]

Smalltalk about the baby and the current situation…

Background/demographic information

Please ask for the woman's age and how many pregnancies and childbirths she has had before. How many of her children are alive? Any particular story to tell?

Where did you give birth last time?

Where do you live in relative to your birth family? How often do you see/visit them?

How far is it to the nearest health facility? *[< 1 hour walk, 1-3 hours walk, > 3 hours walk]*

Antenatal care

Thinking back to when you were pregnant:

Can you tell me about any antenatal care you received during pregnancy? What was your experience of this care? *[what did they do, where did you go, how often, was this a positive/negative experience. If not, why? Did you pay out of pocket?]* What advice did you receive at this point about where you could give birth?

Did you experience any problems during your pregnancy? *[what kind of problem, what did they do - if she went to the health facility, who did they see, what did they advise]*

If yes, who observed this and were other measures initiated for you? Can you elaborate on that? *[The midwife observed it, she referred me to a higher level of care, she helped/did not help me here in the village, other persons in the village helped me/ evt. who?]*

Where did you plan to give birth? Did you make any specific plans around place of birth? *[putting aside money, asking for support/transport, advice from the midwife]*

What influenced this decision? *[probes: family, previous experience, midwife, health professional, others]*

Who did you discuss this with? *[midwife, husband, mother in law, family member, health professional]*

Where those around you supportive of your views and decisions?

Experience of home birth

Why did you choose to give birth at home? *[access to a midwife to assist me/one-to-one contact, no health facility available, not aware of the health facility, it is too far, no transport, too expensive, perception of poor care, have given birth at home without complication before or given birth at the facility and had a bad experience, heard bad/sad stories from others]*

At the time of birth, who assisted you? *[midwife, doctor, family, traditional birth attendant, other]* Were you happy with this care and support? What did those people do? How did you feel in that situation? *[I felt safe, I did not trust her, She was kind to me, I was afraid.. probe: why? Why not?]*

Would you say your birth was straightforward or did you face any difficulties or complications? *[details]* Can you elaborate on that?

Can you tell me a bit about what happened after you gave birth? *[immediate contact with baby, any problems with baby, support to breastfeed]*

What do you think are the advantages and/or disadvantages of giving birth at home?

Have you or your baby experienced any health problems since the birth? What did you do? Who do you ask for advice?- and why this person?

Have you made any visits to the health facility since giving birth? *[postnatal check, vaccinations; who provide]* If not, has your baby had any health care since being born? *[an outreach clinic, a traditional "doctor", none]*

How would you describe the quality of the care/support you received overall? *[good and bad parts of care]* Why?

Experience of the health facility

If the woman has had no contact with a health facility, it will not be possible to get answers for all of the following questions. You should continue in order to gage her awareness of the facilities activities and her reasons for not using it

Can you tell me about any specific interactions you've had with the health facility (in last year)? *[what kind of interaction, who saw her, frequency, quality]* Have any of these involved a midwife?

If yes, what was/is your general impression of the health facility? *[appearance, atmosphere, friendliness, accessibility, staff]* Do you think your experience of the health facility influenced your decision about where to give birth?

If no, have any friends or family used the clinic? *[what kinds of interaction, who did they see]* Did they tell you their opinion of the facility? *[negative/positive]* Did you consider this persons opinion when choosing where to give birth?

Do you know anything about the midwives at the health facility? What do you think the main job and responsibilities of a midwife are? What role do you think they have in improving women's and children's health? We would be interested to know anything you know about the midwives and the care they provide.

What do you think are the advantages and disadvantages of giving birth at a health facility?

What is your experience of giving birth in a facility?

Closing questions

When you look back on your care during pregnancy, at birth and since birth, do you feel happy with the care you have received? *[why, tease out elements]*

Can you tell me about anything you would like to see improved?

If you were having another child would you like to change anything? *[either about where you would choose to give birth or about the care you received]*

Where would you recommend your sister or friend to give birth? Why? Who would you advice her to assist her when in labour?

Do you think in general that the care women receive during pregnancy and birth has improved over time or do you think there has been little change, for example in the last

three years? Do you think the service has changed since the midwife started working in your village?*? [explore in what way]*

Is there anything else/a story you would like to tell me about your experience of pregnancy, giving birth or any interaction with the health facility and care from the midwives there?

Are there any questions you would like to ask me?

Interview guide: Focus Group Discussions (FGDs)

Discuss what they know and have understood about the study, its implications and improvement of health care service in the area. Understanding the structure of health care, health challenges and traditions in the village in general and maternity health in particular. Understand the knowledge and perceptions of professional health care, its benefits and eventual drawbacks *(could you tell me about.., how do feel about... What does it mean ....and can you tell me a little about it.)*

Understanding the local women´s perceptions, beliefs and attitude toward a professional health care provider in general, and the midwifery service in particular *(can you tell what happen if ..., could you tell me about.., how do feel about... What understanding do you have of., can you tell me a little about it... and what do you think)*

Understanding the challenges, barriers, issues and needs of professional midwifery care *(what is the challenge and barrier...., could you tell me about.., how do feel about... What is the value of..., can you tell me how....do you have a story to tell)*

Understanding the quality of maternity care, the awareness of good health and early detection of complications *(can you tell what happen if ..., could you tell me about.., how do see.., how current health service can be optimized... what is your practice.., what do*

you do..., why...)

Let them tell stories from the village- positive and negative- around pregnancy and childbirth *[What happened? Why do you think it happened? What did you do/not do? Why? Why not?]*

Interview guide: Midwives

Background/demographic information

Ask about the midwife´s age, how long she has been working as a midwife and how long she has been working in this village. How many pregnancies and childbirths has she attended the last six months?

How far is it to the nearest health facility? *[< 1 hour walk, 1-3 hours walk, > 3 hours walk]*

Antenatal care

Can you tell me about the antenatal care service (ANC) in this village? How often do you see women for ANC? What is your experience of this care? *[what do you do, where do you perform it, how often, is this a positive/negative experience of your work? If not, why?]*

What is your experience of this care? *[the women appreciate it, I get to know the women, I notice discrepancies in pregnancy, I learn about the pregnancy, it is nice…]*

Did you experience any problems during this service? *[what kind of problem, women do not come, other…]*

Experience of home birth

Thinking back to some of your last home deliveries… Why did you choose to deliver the baby at home? *[no health facility available, not aware of the health facility, it is too far, no transport, too difficult to get there, perception of poor care, have attended home births without complication before, have bad experiences from the health facility]*

How did you feel in that situation? *[I felt safe, confident, I knew the woman well, I was afraid.. probe: why? Why not?]*

Who helped you during the labour and childbirth- or did you perform everything alone? *[the mother in law, other family member, traditional birth attendant]*

Was the delivery straightforward or did you face any difficulties or complications? *[details]* Can you elaborate on that? *[tell about complications, problems with transport/infrastructure, security problems to get to the woman, other]*

How do you deal with this kind of challenges? *[ask for help from family members, referral of the woman, manage to solve the problem, do not know what to do…]*

How do you feel when you experience these challenges? *[Sad, angry, frustrated, crying, happy to manage, proud)*

What happened afterwords? *[immediate contact with baby, any problems with baby, support to breastfeed, peace and happiness]*

What do you think are the advantages and/or disadvantages of giving birth at home?

Have you experienced any problems with the woman postpartum? What did you do? Who do you ask for advice?- and why this person?

Have you visited the woman postpartum? *[general postnatal check, check for the baby, vaccinations; other]* If not, why?

Experience of the health facility

Can you tell me about any specific interactions you've had with the health facility (in last year)? *[what kind of interaction, frequency of referrals, quality]* Have any of these involved another midwife?

What was/is your general impression of the health facility? *[appearance, atmosphere, friendliness, accessibility, staff]* Do you think your experience of the health facility influenced your decision about advice/referring women to the facility?

Have any friends or family of you used the clinic? *[what kinds of interaction, who did they experience]* Did they tell you their opinion of the facility? *[negative/positive]* Did you consider this persons opinion when you advice women where to give birth?

What are the advantages and disadvantages of giving birth at a health facility?

Closing questions

What do you suggest to improve the conditions for women and children in pregnancy and childbirth in the village?

How do you think you can achieve this?

Do you think in general that the care women receive during pregnancy and birth has improved over time or do you think there has been little change, for example in the last three years?

Do you think the service has changed since you as a midwife started working in the village? *[explore in what way]*

Do you want to tell a story (good or bad) from your practice as a midwife underlining the challenges you are facing?

Are there any questions you would like to ask me?
